# HPTLC-Bioluminescent Bioautography Screening of Herbal Teas for Adulteration with Hypolipidemic Drugs

**DOI:** 10.3390/bios13030392

**Published:** 2023-03-17

**Authors:** Yuting Wang, Xingjun Xi, Liao Wang, Yisheng Chen

**Affiliations:** 1 State Key Laboratory of Food Science and Technology, Nanchang University, Nanchang 330047, China; 2 Sub-Institute of Agricultural Food Standardization, China National Institute of Standardization, Beijing 100191, China; 3 School of Food Science and Technology, Jiangnan University, Wuxi 214122, China; 4 College of Food Science and Engineering, Shanxi Agricultural University, Taigu 030801, China

**Keywords:** HPTLC, bioluminescence, herbal tea, hypolipidemic activity, adulteration

## Abstract

Teas based on nutraceutical herbs are an effective tool against hyperlipidemia. However, the adulteration with chemical drugs is frequently detected. By coupling bioluminescent bioautography with high performance thin-layer chromatography (HPTLC), we developed a facile method suitable for screening hypolipidemic drugs (ciprofibrate and bezafibrate) adulteration in five different herbal teas (lotus leaf, Apocynum, Ginkgo biloba, Gynostemia and chrysanthemum). First, the sensitivity of a bioluminescent bacteria to the analyte was evaluated on different HPTLC layer materials, revealing that the best performance was achieved on the silica gel layer. On this basis, sample extracts were separated on silica gel plates via a standardized HPTLC procedure, forming a selective detection window for the targeted compound. Then, the separation results were rapidly visualized by the bioluminescence inhibition of bacteria cells within 6 min after dipping. The observed inhibition displayed an acceptable limit of detection (<20 ng/zone or 2 mg/kg) and linearity (*R*^2^ ≥ 0.9279) within a wide concentration range (50–1000 ng/zone). Furthermore, the optimized method was performed with artificially adulterated samples and the recovery rates were determined to be within the range of 71% to 91%, bracing its practical reliability. Showing superiorly high simplicity, throughput and specificity, this work demonstrated that the analytical method jointly based on HPTLC and bioautography was an ideal tool for screening bioactive compounds in complex biological matrix.

## 1. Introduction

Hyperlipidemia is a metabolic disease characterized of abnormally increasing total cholesterol, low-density lipoprotein cholesterol and triglyceride levels or decreasing high-density lipoprotein cholesterol. Hyperlipidemia has been a highlighted risk factor for public health since it is closely related to cardiovascular diseases and is the major cause of morbidity and mortality world-widely. Remarkably, the prevalence of lipid metabolism disorder is rapidly increasing not only in the elderly people, but also in the adolescent, which may be attributed to the high-oil dietary and irregular lifestyle. The modern pharmacological medication is an effective way to maintain normal lipid profile but is associated with many side-effects. 

In this regard, marked attention was paid to the health tea based on nutraceutical herbs. It has been experimentally demonstrated that phytochemicals from many nutraceutical herbs had hypolipidemic activities by modulating various molecular targets and related pathways [1,2,3,4]. For example, Weng reported that the dammarane-type glycosides from Gynostemma showed promising hypolipidemic activity by inhibiting proprotein convertase subtilisin/kexin type 9 in HepG2 cells [5]. The hypolipidemic effects of hot water leaf extract was also experimentally evidenced [6].

More importantly, these nutraceutical herbs have a long history as food stuff, guaranteeing their safety as important ingredients of health tea. As hypolipidemic active nutraceutical herbs have become increasingly popular in the health food markets around the world, the adulteration with synthetic potent drug poses a serious challenge to food safety agencies. Not surprisingly, this has been a common problem of herb products. For example, many “all natural” herb product claimed to improve sexual performance have artificially added a 5-phosphodiesterase inhibitor that is originally used as a relaxing cardiovascular smooth muscle drug [7]. 

Many methods had been proposed for authenticating herbal tea [8,9]. In contrast to the “gold method” based on column chromatography, high performance thin-layer chromatography (HPTLC) gained remarkable attention. Conventionally, HPTLC was underestimated for its relatively poor separation and low sensitivity. Nevertheless, the unique merit of its compatibility was highly acknowledged [10,11,12,13,14]. Apart from that, HPTLC also displayed many intrinsic advantages, such as simplicity, matrix-tolerance and high-throughput. This enabled the analyst to expand the capacity of HPTLC by easily integrating a large array of assays that conventionally performed interpedently, such as surface enhanced Raman spectroscopy [15,16,17] and IR [18,19]. Particularly, HPTLC separation in combination with elaborately chosen effect-directed assays, also termed bioautography, had been demonstrated a novel and promising tool for screening of compounds with specific bioactivity in bio-mixtures [20,21,22,23].

In this work, a selective and reliable HPTLC method for screening two hypolipidemic drugs, including ciprofibrate (CPF) and bezafibrate (BZF), was developed with a sensitive bioautography based on the response of microbial bioluminescence, which was completely different from conventional chemical derivatization. In addition, the established method was further validated with different herb extracts to evidence its applicability.

## 2. Materials and Methods

### 2.1. Chemicals and Instruments

The reference standard of ciprofibrate (purity ≥ 99%, HPLC) and bezafibrate (purity ≥ 96.0%, HPLC) were purchased from Aladdin (Shanghai, China). Na_2_HPO_4_, KH_2_PO_4_, glycerol and other reagents with analytical purity was purchased from Sigma-Aldrich (Shanghai, China). Peptone and yeast extract were from Sinopharm (Beijing, China). A Millipore Synergy system (Schwalbach, Saarland, Germany) was used to prepare Ultra-pure water. A HPTLC work station, including a semi-auto sampler Linomat 5, an automatic developing chamber ADC2 and biovisualizer was from CAMAG (Muttenz, Basel, Switzerland). Ultrasonic water bath (CSSY-80) was from Yichen (Jintan, Jiangsu, China).

Six different glass backed plates was used: (1) silica gel F_254_ plates (analytical grade, 10 cm × 20 cm, serial No. 1.05729.0001) were from Merck (Darmstadt, Germany); (2) NH_2_ bonded silica gel F_254_ plates (analytical grade, 10 cm × 20 cm, serial No. 811111) were from MN (Düren, Bayern, Germany); (3) the silica gel plates (10 cm × 20 cm), Neutralized aluminum oxide plates (10 cm × 10 cm), Acidified aluminum oxide plates (10 cm × 10 cm) and Diatomite plates (10 cm × 10 cm) were from Qingdao Haiyang (Qingdao, Shandong, China). Five authentic health teas based on lotus leaf (LL), Apocynum (AC), Ginkgo biloba (GB), Gynostemia (GS) and chrysanthemum (CT) were purchased from the local market.

### 2.2. Preparation of Bioluminescent Suspension

**Figure 1:**
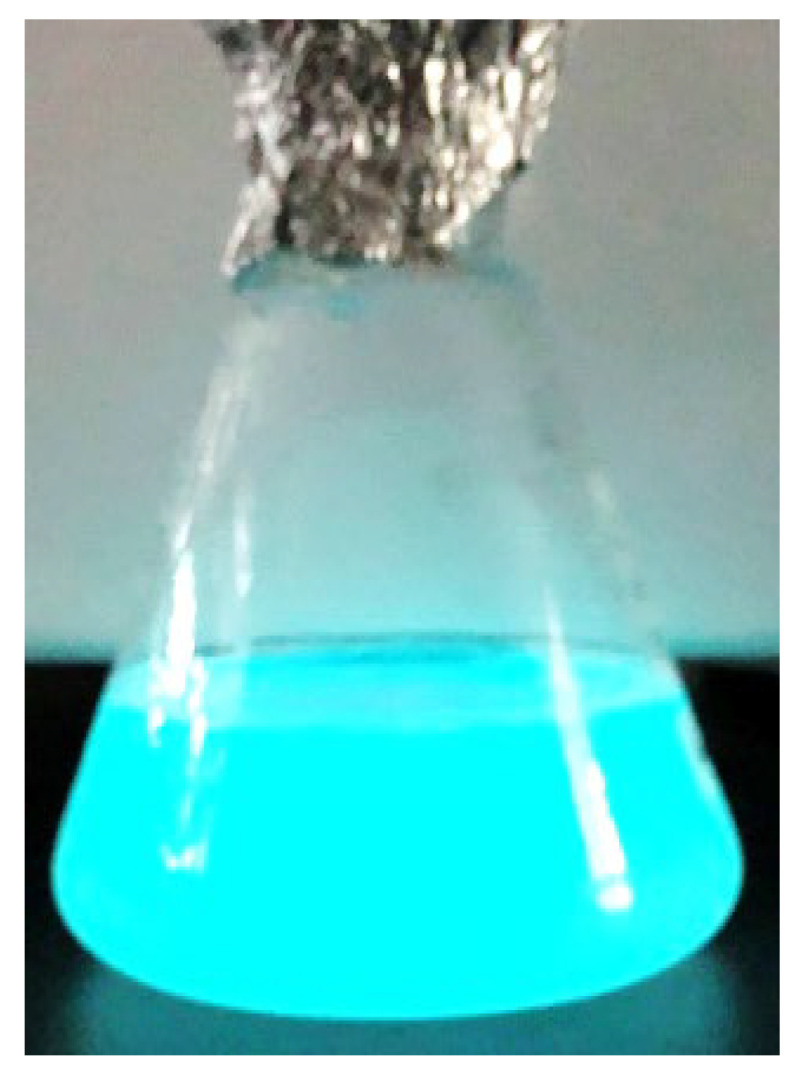
Bioluminescence of the bacteria suspension.

The bioluminescent bacteria strain *Photobacterium phosphoreum* was provided by Nanjing Institute of Soil Science, Chinese Academy of Sciences. The method of preparing bacterial suspension with bioluminescence was principally based on the steps previously described [23,24]. When strong greenish bioluminescence became observable to eye inspection, as shown in Figure 1, the bacterial suspension was diluted with another 100 mL liquid medium prior to usage.

### 2.3. Preparation of Standard Solution

An amount of 10 ± 0.1 mg reference standards of CPF and BZF were added into a 10 mL volumetric flask, respectively. The flask was filled with 10 mL methanol, resulting in 1 mg/mL standard stock solution. The working solution was prepared by further diluting the stock solution to 0.02 mg/mL.

### 2.4. Preparation of Tea Samples 

The dried tea samples were smashed to powder. An amount of 1 g sample powder was mixed with 10 mL methanol. The mixture was conditioned in ultrasound bath at 25 °C for 30 min, then centrifuged at 5000 rpm for 5 min. An amount of 5 mL of the obtained supernatant was evaporated and recovered with 0.5 mL methanol. Then, the extract was filtered through 0.45 μm nylon membrane for further analysis.

### 2.5. HPTLC Steps

With a Linomat 5 semi-automatic sampler, solution of standards and samples extract (10 uL) were sprayed as 6 mm bands, facilitated by a 0.5 MPa nitrogen stream. Dosage speed of spraying is 100 nL/s, with predosage volume at 0.2 nL. The band array was 10 mm from the plate bottom and at least 15 mm from both sides. 

After evaporating the sample solvent in the application band, chromatography was performed in the ADC-2 automatic developing chamber using ethyl acetate+methanol (9 + 1 mL) as the mobile phase with constant settings: 3 min humidity control with saturated MgCl_2_, 10 min chamber saturation, 10 min plate pre-conditions and 60 mm migration distance. Then, the mobile phase residue was completely evaporated at 80 °C for 5 min. Pictures of the separation results on the HPTLC plate was documented by a HPTLC image system DD70, illuminated by 254, 366 nm and with light, respectively.

### 2.6. Documentation and Analysis of the Bioluminescent Image

After chromatography, the dried plate was dipped into the prepared bacteria suspension with strong bioluminescence by a TLC immersion Device 3, with constant settings: dipping speed 1 mm/s and staying time 2 s. Then, the plate was immediately placed into the sample chamber of a biovisualizer. Then, documentation of bioluminescence images was saved in black/white and colorful format, respectively. The bioluminescent inhibition profile was quantitative evaluated by Videoscan, respectively, based on the grayscale value of pixels. 

## 3. Result and Discussion

### 3.1. Optimization of HPTLC Layer Material

The spectroscopic character of the targeted analyte of this study was poor, neither with nature fluorescence nor fluorescence quenching. Therefore, they were hardly visible on the HPTLC plate, necessitating derivatization. Different from conventional chemical derivatization reactions, bioautography based on whole cell displayed strong dependence on layer materials, which had been experimentally evidenced in the HPTLC analysis of antibiotics [25] and alkaloid [26]. Such layer-induced sensitization can be employed to strength the detectability of analyte. In this regard, we evaluated the bioautography results of the analyte deposited on five most used HPTLC layer materials, including silica gel, NH2-silica gel, acidified/neutralized aluminum oxide and diatomite. After dipping, the bioluminescence from the bacteria differed dramatically, as comparatively summarized in Table 1. Plate layers made of aluminum oxide and diatomite were demonstrated to be not suitable for the used bioautography because their exposure strongly inhibited the bioluminescence. Meanwhile, brilliant background can be observed both on silica gel and NH_2_-silica gel. However, sensitive inhibition spots down to 20 ng/zone can be observed on the silica gel, while no inhibition spot was detected on NH_2_-silica gel. Therefore, the F_254_ silica gel plate was used for further study.

### 3.2. Optimization of Bioluminescent Bioautography

**Figure 2:**
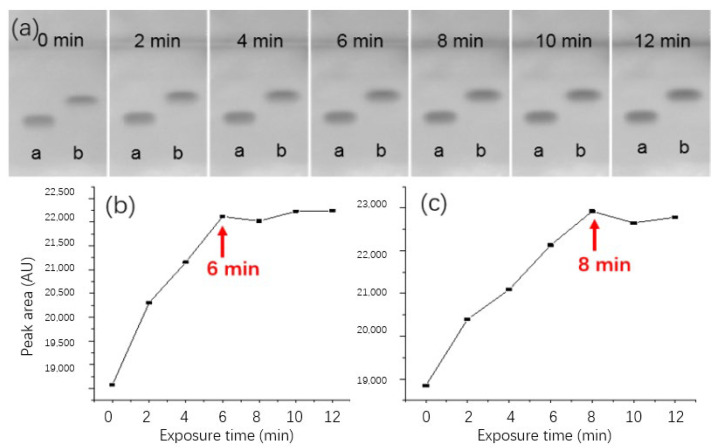
(**a**) The change of bioluminescent inhibition pattern within the initial 12 min after dipping. Track assignment: a—BZF, b—CPF; corresponding peak area variation profile for (**b**) BZF and (**c**) CPF.

Different from conventional derivative reactions, the targeted molecule must penetrate the membrane of bacterial before its cytotoxicity can be sensed. Therefore, the bioluminescence inhibition pattern showed strong time-dependence in the initial few minutes. Apart from that, it must be kept in mind that the area of inhibition zone also increased along with exposure time due to diffusion. To fix an optimal balance between detectability and resolution, we quantitatively evaluated the change of inhibition profiles in the initial 12 min of exposure. As shown in Figure 2a, weak inhibition spots can be observed immediately after exposure. Then, the inhibition strength increased rapidly along with incubation time. Figure 2b–c further analyzed the change quantitatively, from which it was clear that the intensity of inhibition caused by BZF reached a platform stage after 6 min exposure, while the time for CPF was 8 min. Thereafter, signal intensities changed insignificantly, while zone diffusion became apparent. Considering these results, pictures were documented at 8 min after dipping for further experiments. 

### 3.3. Optimization of Chromatographic Conditions 

**Figure 3:**
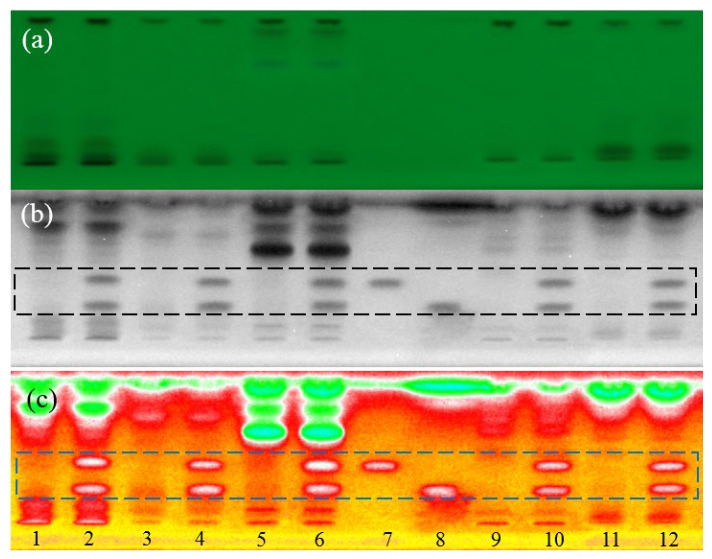
Images of a developed plate under different conditions: (**a**) underivatized-254 nm light, (**b**) dipped into bacterial suspension-bioluminescence inhibition (black/white mode) and (**c**) dipped into bacterial suspension-bioluminescence inhibition (color mode). Track assignment: 1 LL, 2 spiked LL, 3 AC, 4 spiked AC, 5 GB, 6 spiked GB, 7 CPF standard, 8 BZF standard, 9 GS, 10 spiked GS, 11 CT and 12 spiked CT; mobile phase ethyl acetate + methanol (9/1, *v*/*v*), standards concentration 150 ng/zone.

The complexity of the targeted samples raised a serious challenge to the selectivity of detection. In order to prevent background interferences due to co-extractants, chromatographic conditions were optimized before bioautography. First, a trail of mixtures containing methanol, ethanol and ethyl acetate were tested for the separation. The selection of mobile phase was based on two principles: viscosity and toxicity being as low as possible. After comparison, it was found that the mixture of ethyl-acetate+methanol (9 + 1 mL) gave a preferable window of the targeted compound; in contrast, most endogenous substances that were not fully visible to 254 nm light were pushed upward, as shown in Figure 3a–c. This enabled straightforward identification of possible adulteration. Moreover, semi-quantitative data can be estimated simply by eye inspection, which was greatly suitable to screening tasks. 

### 3.4. Precision and Sensitivity

**Figure 4:**
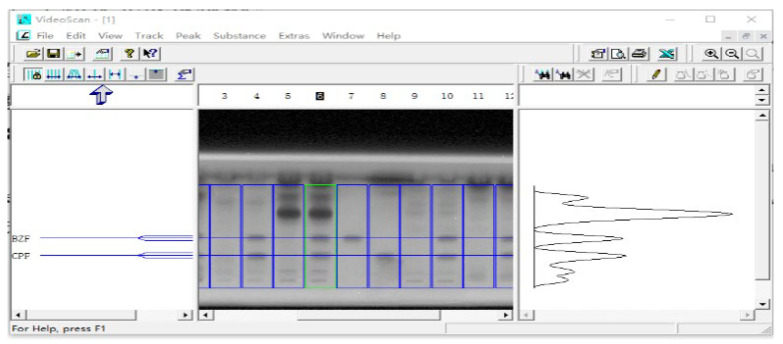
Quantitative analysis of the bioluminescence inhibition spots in HPTLC image by Videoscan software.

As an alternative to straightforward eye inspection, evaluation of the digital image by software enabled a more precise analysis of the bioautographic results. This was principally based on virtually converting the grayscale pixel to chromatogram. Thus far, there had been a couple of software available to extract quantitative data from digitalized HPTLC images [27]. In this study, the software Videoscan tailored for analyzing HPTLC images were used for obtaining the integration result of chromatographic peak, which was exemplarily shown in Figure 4.

From the pixel analysis, it was revealed that the baseline drifting of the chromatogram was usually more serious than that of chemical derivatization. Therefore, the precision of detection was assessed first. From the obtained data, it was revealed that the overall RSD% of detection was <11.8% and therefore acceptable for screening analysis. In order to determine the limit of detection of the established method. Analyte zones at gradient concentrations from 25 to 5 ng/zone was assessed. Noticeably, the inhibition signal caused by the analyte did not display linear at concentrations <20 ng/zone. More specifically, spots of the analyte at 20 ng/zone were still visible. However, if the concentration was reduced to the 15 ng/zone, the inhibition became invisible. In another word, the sensitivity of this method was >20 ng/zone. Taking the application volume into consideration, this detectability was equal to 2 mg/kg.

### 3.5. Linearity and Accuracy

**Figure 5:**
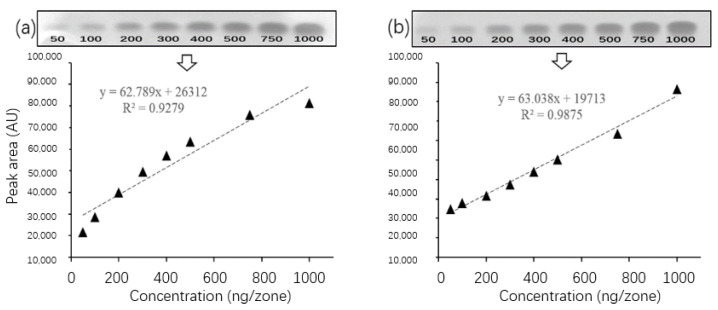
Does-dependence profile of inhibition zones caused by the analyte from 50–1000 ng/zone: (**a**) BZF and (**b**) CPF.

Based on the optimized conditions, we further evaluated the signal–concentration relationship within the critical concentration range 50–1000 ng/zone. As shown in Figure 5, the chromatographic signals transformed from pixel grayscale values conformed dose-dependence with coefficient of determination R^2^ ≥ 0.9279, suggesting that the developed method was acceptable for quantitatively screening. In order to access the accuracy of analysis, blank samples with artificial adulteration were measured. As internal standard, three levels of the analyte at concentrations at 5 mg/kg, 10 mg/kg and 20 mg/kg, respectively, were spiked into the sample extraction mixture. As summarized in Table 2, the calculated recovery rates for all spiked extracts were within the range of 71% to 91%, showing little dependence on the sample matrices. These quantitative results conclusively evidenced that the established method could be a robust tool for screening the analyte in herbal tea samples.

## 4. Conclusions

In this work, a fast and facile HPTLC method was developed for the screening of chemical adulteration in herbal tea with hypolipidemic activity. First, the bioluminescent assay was optimized with different HPTLC layer materials. Then, the strong background noises due to co-extracted sample matrices were prevented by separation on HPTLC plate. Under optimization of chromatography and bioautography conditions, the developed method gave an acceptable limit of detection and linearity (R^2^ ≥ 0.9279) within a wide concentration range (50–1000 ng/zone). Meanwhile, the validation with real samples suggested that this method had enough accuracy (spike-recover rate within 71% to 91%). This work demonstrated that HPTLC was a promising platform for the effect-direct assay, which was able to greatly simplify the screening and identification of bioactive compounds in food.

## Figures and Tables

**Table 1:** Characterization of the effects of layer materials on the detectability. Note: Images were from plates without development after 8 min exposure to bacteria suspension.

Group 1						
Layer materials	NH_2_-Silica gel F_254_ 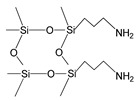		Silica gel F_254_ 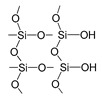		Silica gel 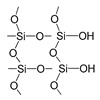	
Exposure time (min)	0	8	0	8	0	8
Image	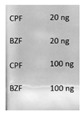	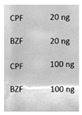	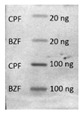	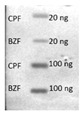	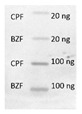	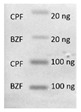
Usability	NO		YES		YES	
**Group 2**						
Layer materials	Neutral aluminum oxide Al_2_O_3_		Acidified aluminum oxide Al_2_O_3_		Diatomite SiO_2_	
Exposure time (min)	0	8	0	8	0	8
Image	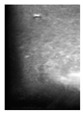	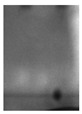	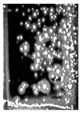	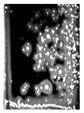	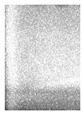	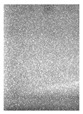
Usability	NO		NO		NO	

**Table 2:** Accuracy evaluation of the established method.

Analyte	Spiked Levels (mg/kg)	Recovery Rate (%) *				
		LL	AC	GB	GS	CT
BZF	5	81 ± 7	79 ± 6	86 ± 8	80 ± 5	85 ± 5
	10	90 ± 8	89 ± 9	85 ± 7	78 ± 6	71 ± 4
	20	82 ± 8	84 ± 5	88 ± 8	72 ± 6	78 ± 7
CPF	5	74 ± 7	78 ± 7	83 ± 5	83 ± 7	85 ± 6
	10	89 ± 7	90 ± 9	83 ± 6	87 ± 6	81 ± 7
	20	78 ± 3	83 ± 8	91 ± 8	85 ± 7	90 ± 8

* The RSD% was calculated from three duplicates.

## Data Availability

The data could be available on request.
